# Wave after wave: The suggestibility of noise in the experience of multisensory hallucinations under multimodal Ganzfeld stimulation

**DOI:** 10.1177/20416695251376600

**Published:** 2025-09-23

**Authors:** Eleftheria Pistolas, Liv Smets, Johan Wagemans

**Affiliations:** 1 University of Leuven (KU Leuven), Belgium; 2 Catholic University of Louvain (UC Louvain), Belgium; * Eleftheria Pistolas has been awarded the Early Career Best Paper Prize for her work on this article.

**Keywords:** audition, color, binocular vision, imagery, lightness/brightness, light, multisensory/cross-modal processing, perception, visuoauditory interactions

## Abstract

A multimodal Ganzfeld (MMGF) consists of homogeneous stimulation in both the visual and auditory modalities. Exposure to this unique perceptual environment can elicit the awareness of hallucinatory percepts. The nature of these hallucinatory percepts, and specifically the frequency of visual, auditory and multisensorial hallucinations, remains unclear. In this study, an MMGF refers to the stimulation paradigm itself. The perceptual experiences elicited, however, can be unimodal (occurring in one modality), multisensory (simultaneous but thematically unrelated across modalities), or multimodal (thematically integrated across modalities), allowing us to assess multisensory integration in the MMGF. Employing a multimethod approach in which we combine quantitative and qualitative measures, we conducted three experiments, using a between-subjects design with three noise conditions, that is, no-noise, white-noise, and brown-noise. Experiments 1 and 2 were conducted in a laboratory Ganzfeld (GF) space, Experiment 3 was conducted in a GF art installation in a museum context. We conducted half-open interviews, analyzed using inductive content analysis, to grasp the subjective experience and assess congruency of visual and auditory hallucinations. We found that visual hallucinations were frequently reported, but auditory hallucinations were less common. The most consistently reported auditory hallucinations, and importantly, multisensory integrated hallucinations, were water-related, suggesting a potential influence of noise, particularly brown noise, possibly due to its resemblance to water sounds. Our findings also indicate a predominantly unimodal focus on the visual aspect among participants, alongside instances of attention switching between modalities.

## How to cite this article

Pistolas, E., Smets, L., & Wagemans, J. (2025). Wave after wave: The suggestibility of noise in the experience of multisensory hallucinations under multimodal Ganzfeld stimulation. *i–Perception*, *16*(5), 1–18. https://doi.org/10.1177/20416695251376600

## Introduction

The term Ganzfeld (GF) denotes a homogeneous field of light (Metzger, 1929), devoid of a gradient of luminous intensity, with no transitions between lighter and darker areas (Gibson & Waddell, 1952). Various methods have been employed to establish GF stimulation in past research, ranging from homogeneous spaces (Metzger, 1929), closed eyes viewing of a bright light (Billock & Tsou, 2012), to GF goggles made of ping pong balls cut in half (Schmidt et al., 2020). Preceding GF research, translucent goggles were employed for sensory deprivation experiments, combined with white auditory noise (Heron et al., 1956) until later research reported the emergence of hallucinations increased with diffused stimulation, as the GF provides (Zubek, 1969). The original GF literature has strictly focused on a visual GF (e.g., Avant, 1965; Cohen, 1960; Gibson & Waddell, 1952; Hochberg et al., 1951), however a GF can also be applied to other modalities, such as audition. The simultaneous application of a GF with homogeneous noise is termed a multimodal GF (MMGF; Kübel et al., 2021; Pütz et al., 2006; Schmidt & Prein, 2019), a method that has been used in more recent GF research (Kübel et al., 2021; Lloyd et al., 2012; Pütz et al., 2006; Schmidt et al., 2020; Schmidt & Prein, 2019; Wackermann et al., 2008).

After prolonged exposure to an MMGF, subjects have been found to experience alterations in consciousness (ASC; Glicksohn, 1991; Kübel et al., 2021; Pütz et al., 2006; Schmidt & Prein, 2019; Wackermann et al., 2008). An ASC is defined as “any mental state recognized as representing a sufficient deviation in subjective experience from certain general norms during alert, waking consciousness” (Glicksohn, 1993, p. 2) and is characterized by an altered time perception (Glicksohn, 1992; Kübel et al., 2021; Schmidt & Prein, 2019), reduced self-awareness, relaxation, absence of structured thinking (Glicksohn, 1991; Vaitl et al., 2005), and frequently, the emergence of hallucinatory percepts (Bexton et al., 1954; Lloyd et al., 2012; Miskovic et al., 2019; Pütz et al., 2006; Schmidt & Prein, 2019; Wackermann et al., 2002, 2008).

These hallucinatory percepts can include dreamlike imagery (Miskovic et al., 2019; Pütz et al., 2006), with elements of familiarity (Pütz et al., 2006; Wackermann et al., 2002), and can occur on one (i.e., unimodal) or multiple modalities simultaneously (i.e., multimodal/multisensorial). In terms of visual hallucinations, examples of participants’ experiences under MMGF stimulation comprise changes in color intensity or hue, dots, lines, simple geometric patterns, faces, recognizable figures, and integrated scenes with movement (Bexton et al., 1954; Lloyd et al., 2012; Miskovic et al., 2019; Pütz et al., 2006; Schmidt & Prein, 2019; Wackermann et al., 2002, 2008). On the auditory modality, reports of MMGF experiences can include beeping, alarms, whistles, birds, water, voices, music (Bexton et al., 1954; Lloyd et al., 2012; Miskovic et al., 2019; Schmidt & Prein, 2019). Olfactory, kinesthetic, proprioceptive and tactile hallucinations are less frequently reported in the MMGF (Wackermann et al., 2008). Examples include detachment from the body, weightlessness, feelings of sinking and floating (Bexton et al., 1954; Lloyd et al., 2012; Wackermann et al., 2008). Besides these unimodal hallucinations, multimodal and multisensory hallucinations can also be part of the MMGF experience.

Toh et al. (2022) argue for a framework that describes multimodal and multisensory hallucinations as hallucinations that occur in multiple modalities at the same time. However, a distinction should be made in terms of overlap in thematic content; multimodal hallucinations are related in thematic content, multisensory hallucinations are not necessarily congruent regarding the thematic content. It is not clear whether unimodal GF stimulation can result in multimodal or multisensory hallucinations given that the focal point of these studies was the visual perception aspect and therefore, authors of these studies either did not assess phenomenology in multiple modalities or did not report on it. Despite descriptions of such multimodal phenomena, a significant gap in the literature remains regarding the precise frequency and specific nature of these multimodal hallucinatory experiences under GF stimulation when participants are instructed to report hallucinatory experiences in any sensory modality.

As such, the current state of the literature suggests that multimodal and multisensory hallucinations occur mostly, if not exclusively, under MMGF stimulation, while unimodal hallucinations can occur during both variants of the GF (Bexton et al., 1954; Lloyd et al., 2012; Pütz et al., 2006; Wackermann et al., 2002, 2008). Multimodal hallucinations reflect the emergence of multisensory integration in the MMGF. Unimodal auditory hallucinations have generally been found to be simple, however, when they co-occur with visual imagery and thus resulting in multimodal hallucinations, they have been found to take a more complex form, embedded in a meaningful context (Pütz et al., 2006; Wackermann et al., 2008). In previous research, which employed waterfall sounds as the auditory GF, hallucinatory percepts were found to be water-related (Wackermann et al., 2002). In some cases, multimodal and multisensory hallucinations have also been reported in more than two modalities, such as the visual, auditory and kinesthetic modalities (Wackermann et al., 2008).

Clarity pertaining to the frequency of visual, auditory and multisensorial hallucinations is lacking in the current literature. This study primarily sought to explore the spontaneous multimodal phenomenology and multisensory integration occurring in GF experiences with and without auditory input when participants are instructed to report arising percepts in any sensory modality. While exposure to MMGF stimulation is known to induce hallucinatory percepts, clarity pertaining to the frequency of visual, auditory and multisensorial hallucinations and a detailed qualitative understanding of their nature are lacking in the current literature. In this study, the term “multimodal GF” refers specifically to the stimulation paradigm involving homogeneous input in both the visual and auditory modalities. The perceptual experiences elicited, however, may be unimodal, multisensory (simultaneous but thematically unrelated), or multimodal (thematically integrated across modalities). This distinction allows us to empirically assess the presence or absence of multisensory integration. Addressing this gap, the present study employed a mixed-methods approach across three experiments to provide a more comprehensive quantitative and qualitative understanding of the MMGF experience. We systematically investigated the frequency and content of spontaneously reported visual, auditory, and multisensorial percepts, allowing participants to report experiences in any sensory modality. By including different types of homogeneous noise (white, brown, or no noise), we explored the potential influence of the auditory stimulus. Through quantitative analysis of hallucination frequencies and detailed inductive content analysis of qualitative interview data, our study aimed to clarify the nature and prevalence of unimodal and multisensory integrated (i.e., multimodal) experiences, including potential influences of noise.

In this article, we will focus on the interview data, which captures the multisensory information relevant to our investigation. This study is part of a broader research project with several interconnected aims. Specifically, the project set out: (a) to clarify GF phenomenology and the terminology used to describe these phenomena in past literature, primarily focusing on the visual modality; (b) to study the multisensory and multimodal GF experiences, with a particular emphasis on multisensory integration, which is the central focus of the current paper; (c) to investigate the altered states of consciousness elicited by the GF effect, including coregistration of eye-tracking and EEG data; and (d) to explore the immersive aesthetic experiences artists elicit using the GF effect in their light installation artworks. Experiment 3, conducted in a museum art installation, served to explore the aesthetic responses in a more ecologically valid setting. For reasons of brevity and to maintain the core focus of this article, other data collected as part of this larger project will be analyzed and reported in subsequent papers.

## Method

### Participants

**Figure 1.**
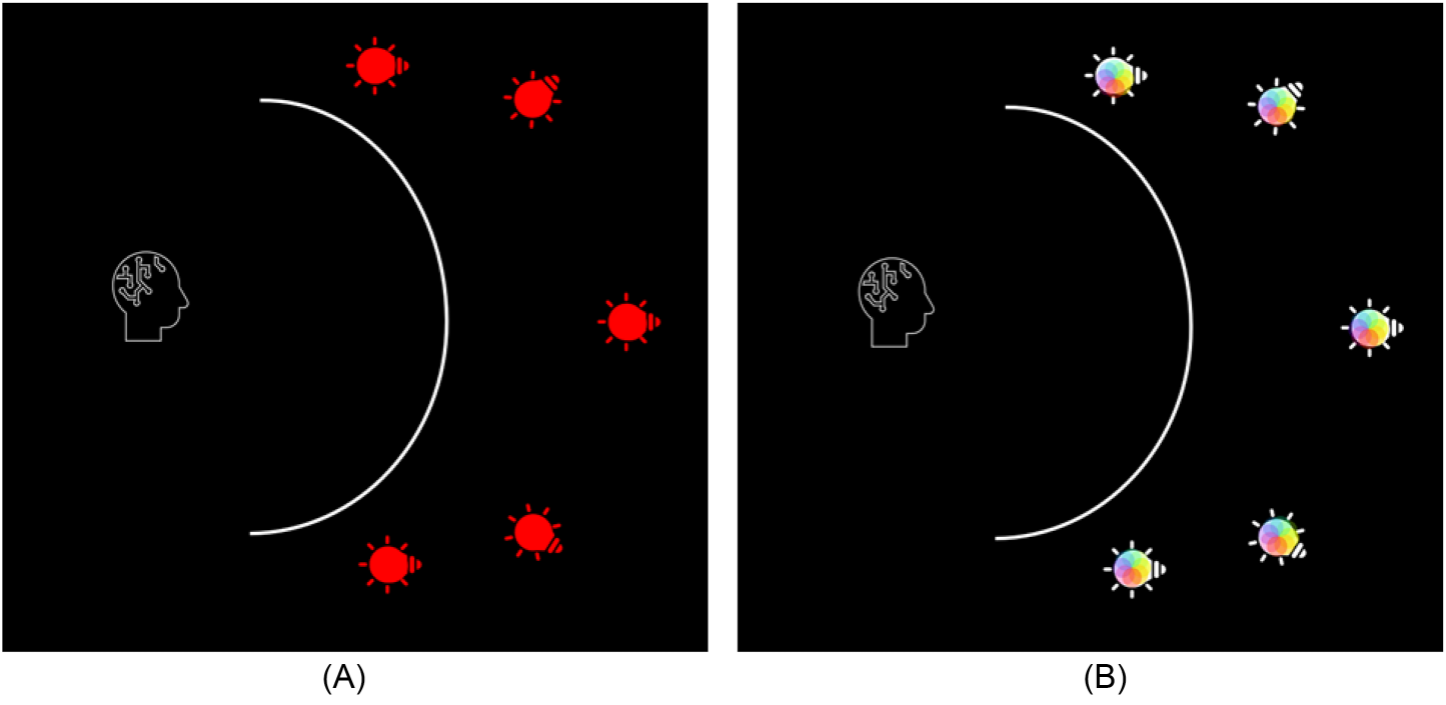
(A) Experiment 1: Observer in front of translucent hemisphere, lit from the outside using five lights that remain red. Figure licensed under CC bY 4.0 by the authors. Retrieved from https://doi.org/10.6084/m9.figshare.27890142.v1. (B) Experiment 2: Observer in front of translucent hemisphere, lit from the outside using five lights that gradually change color. Figure licensed under CC BY 4.0 by the authors. Retrieved from https://doi.org/10.6084/m9.figshare.27890460.v1.

The present study aimed at a better understanding of the MMGF and, in particular, whether and when multimodal integration occurs. In a first experiment, 28 participants were exposed to a red GF in a laboratory setting. In a second experiment, 45 participants viewed the in-lab GF with varying colors. The laboratory set-up used for Experiments 1 and 2 consisted of a translucent curved screen with five Philips Hue Discover Outdoor Floodlights (https://www.philips-hue.com/) behind it as depicted in Figure 1 (Pistolas & Wagemans, 2025).

### Apparatus

Luminance intensity of each light was measured at different locations on the screen using a photometer to ensure a homogenous field. Further specifications about this procedure can be found in our recent preprint (Pistolas & Wagemans, 2025). The color transitions were programed using a custom Python (https://www.python.org/) script using the following colors in this sequence: red, orange, red, purple and blue with each color remaining stationary for 5 min and 15-s color transitions in between. More details can be found in our Python script used to automate the light experience shared on our OSF page.

**Figure 2.**
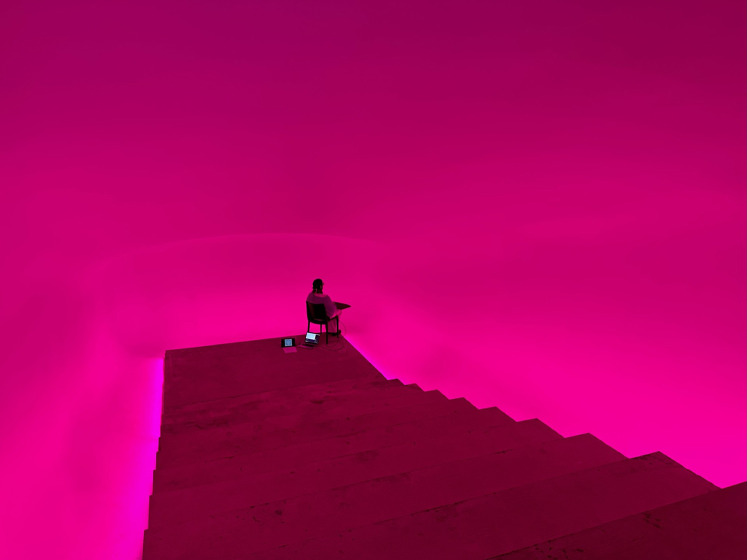
Ganzfeld light installation by Jaap van den Elzen. Figure licensed under CC bY 4.0 by the authors. Retrieved from https://doi.org/10.6084/m9.figshare.27890529.v1.

In a third experiment, we collected data from 67 participants in a GF light installation artwork, created by Dutch artist Jaap van den Elzen at the entrance of the former charcoal mine in Heusden-Zolder (see Figure 2; Van den Elzen, 2022).

All three experiments employed audio as a between-subjects factor with a no-noise, a white-noise and a brown-noise condition. White-noise is a broadband noise with a flat power spectral density across all frequencies, in this case all frequencies of audible sound (Azizi & Yazdi, 2019). Brown-noise has a spectral density proportional to the frequency of the noise. Specifically, brown noise power decreases when frequency increases. More intuitively, brown noise sounds deeper and lower than white noise (Azizi & Yazdi, 2019). The noise files were generated using Audacity 3.2.1 (https://audacityteam.org/). Noise canceling in-ear headphones (Sony LinkBuds S) were used to deliver the noise. Participants were able to adjust the volume to a level that was comfortable for them for 25 min. Ethical approval for this study was granted from the Social and Societal Ethics Committee (SMEC G-2022-5469-R2(AMD).

### Procedure

Participants viewed a GF for 25 min while EEG was recorded using the Smarting Mobi (https://mbraintrain.com/smarting-wireless-eeg/) and eye-movements were registered using the Tobii Pro glasses 3 (Tobii, 2022). Participants were instructed to signal hallucinations and black-outs (and their intensities) using a dial. Preceding the experiment session, participants tried out the dial while looking at a live visualization of the data as they turned the dial. This dial was made using a rotary encoder that is restricted on both sides. The data is read out using Arduino (https://www.arduino.cc/), sent out through USB and recorded using a custom python script. More information can be found in our experiment script and can be accessed through our OSF page.

Early on during data collection, we noticed that the museum context in which Experiment 3 took place was too noisy due to the concrete in the building, despite the noise canceling earbuds, leading to multiple participants reporting being distracted and pulled out of the experience because of the surrounding noise. As a result, we decided to omit the no-noise condition during the remainder of data collection and omit the no-noise data in Experiment 3 for analyses in which we compare conditions because of the discrepancy in number of participants between conditions.

After the 25-min GF session, participants were interviewed and were asked to complete a questionnaire that assessed altered states of consciousness (11-ASC; Studerus et al., 2010), liking and beauty, personality and demographics.

### Questionnaire

The visual analogue scale (VAS) of the 11-ASC was anchored from “No, not more than usual” on the left to “Yes, very much more than usual” on the right. Participants were then asked to report how much they liked the light installation experience (“Did you like the light installation experience?,” 0 = “*no, not at all*,” 10 = “*yes, very much*”) and how beautiful they found the light installation to be (“Did you find the light installation, i.e., what you experienced, beautiful?,” 0 = “*no, not at all*,” 10 = “*yes, very much*”). Seven International Personality Item Pool scales were employed to assess personality (Goldberg et al., 2006), that is, “Artistic interests,” “Curiosity,” “Conscientiousness,” “Attention to emotions,” “Creativity,” “Imagination” and “Spirituality.” In addition, predisposition to hallucinations was assessed using the Launay–Slade Hallucination Scale (Launay & Slade, 1981). The demographical questions assessed age, gender, art interest, whether they have any experience with meditation techniques, whether they have ever tried any hallucinogenic drugs and whether they have ever been diagnosed with a neurological or psychiatric illness and what the diagnosis was.

### Interviews

During the half-open interviews,^1^ participants were asked about their experience (“How was your experience?”), what they saw (“What did you see? This can be anything you have visually seen: images, objects, people, animals, patterns, etc.”), what they heard (“What did you hear? This can be anything you have heard: sounds, voices, music, etc.”), what they smelled (“What did you smell?”), what they felt (“What did you feel in terms of bodily sensations?”), how they felt (“How did you feel, what sort of emotions did you experience?”), what the atmosphere was like (“How was the atmosphere?”), and whether they want to share anything else (“Do you want to share anything else about your experience?”). Lastly, participants were shown a graph in which their dial data was presented. Looking at this graph, we asked them to try to remember the nature and modality of those hallucinations (“In this graph you can see your reports of hallucinations during the experience. Can you try to remember what you saw, heard, smelled or felt in these moments?”). Audio was recorded during the interviews, as disclosed in the consent form. Before the start of data collection for Experiment 3, we decided to add more questions to assess the multimodality of the experience, and potential multisensory integration, better as it became evident that auditory hallucinations occurred less frequently than visual ones. In the case that a participant did not experience any auditory hallucinations, they were asked whether they could think of a potential reason why they would not experience any (“Why do you think you did not experience any auditory things?”). If they answered they did not know, they were asked: “Were you focused on a different sensory modality?” In the case that they did report auditory hallucinations, they were asked (“Were those sounds accompanied by visual things?”). In case the sounds were accompanied by visuals, they were asked: “By what kinds of visual things were the sounds accompanied?” If that was not the case, they were asked: “Do you feel as though your focus or attention switched between sensory modalities?.”

### Qualitative Inductive Content Analysis

We analyzed the qualitative interview data using an inductive content analysis approach. After translating all responses into English, we performed a separate analysis for each interview question (e.g., Visual hallucinations, Auditory hallucinations, Multisensory hallucinations). This bottom-up method involved reading transcribed responses, identifying recurring experiences to form categories, and then coding responses according to these emergent categories. This ensured that our categories were derived from the dataset, rather than focusing on preexisting theory. Our analysis followed a structured process: (a) Familiarization: gaining an overview by reading all responses; (b) Coding: assigning descriptive codes to topics and experiences. It is important to note that a single participant could report multiple distinct hallucinations, and a single description of an experience could be coded into multiple categories if its content spanned several themes; and (c) Synthesis: merging related codes into broader categories. We utilized a flat coding structure, treating all codes with equal importance. The process was iterative, allowing for the emergence of new categories and subsequent recoding as more data was analyzed. To ensure reliability, two researchers independently coded the data, and their codes were compared and reconciled through discussions to finalize the response categories. In the results tables for the inductive content analysis, the numbers presented indicate the number of participants who reported at least one experience that was classified within that specific category.

Table 1 visualizes the measures included in the experiments. All data analyses were conducted using the statistical program R (R Core Team, 2024) and the following packages: dplyr (Wickham et al., 2023), tidyverse (Wickham et al., 2019) and rstatix (Kassambara, 2023).

## Results

The sensory modalities of interest in this article are the visual and auditory sensations. Although some participants reported other sensations, such as bodily sensations of imbalance, these sensations did not occur frequently and have not been the focus of past research either. The number of participants who reported visual hallucinations counted 26(96%), 40(89%) and 63(94%) for Experiments 1, 2 and 3, respectively. Fewer participants reported auditory hallucinations with 7(26%), 8(18%) and 17(25%) for Experiments 1, 2, and 3, respectively. Out of these participants, the ones who experienced multisensory integration, meaning that the perceived imagery was congruent in both the visual and auditory modalities, rendering an integrated whole, counted 2(7%), 2(4%) and 9(13%) in Experiments 1, 2, and 3, respectively. Tables 2, 3, and 4 show the kinds of visual phenomena that came forth out of the inductive content analysis we conducted for Experiments 1, 2, and 3, respectively.

**Table 1.** Overview of measures administered during the experiments.

Measure type	Instrument/question	Scale/format	Reference/notes
Altered states of consciousness*	11-ASC Questionnaire	Visual Analogue Scale (VAS); anchors: “no, not more than usual” to “Yes, very much more than usual”	Studerus et al. (2010)
Aesthetic evaluation*	"Did you like the light installation experience?" "Did you find the light installation beautiful?"	0 = “*no, not at all*” to 10 = “*yes, very much*”	Self-report VAS
Personality traits*	IPIP Subscales: Artistic interests, curiosity, conscientiousness, attention to emotions, creativity, imagination, spirituality	Likert-scale IPIP format	Goldberg et al. (2006)
Hallucination proneness*	Launay–Slade Hallucination Scale-Revised (LSHS-R)	Standard LSHS-R format	Launay and Slade (1981)
Demographics	Age, gender, art interest*, meditation experience*, hallucinogen use*, neurological/psychiatric diagnosis*	Categorical/open-response	Custom questions
Interview questions	How was your experience? What did you see/hear/smell/feel? How did you feel? How was the atmosphere? Anything else to share?	Semi-structured open-ended interview	Audio-recorded (with consent)
Graph-based recall	"Can you try to remember what you saw, heard, smelled or felt in these moments?” (shown personal dial graph)	Follow-up memory prompts based on individual dial data	Added during interview
Auditory hallucination probes	Follow-ups depending on auditory experience: —If none: “why?” and “focused on other modality?” —If yes: “Accompanied by visuals?,” etc.	Conditional branching questions to assess multisensory integration	Added to better capture multimodality

*Note.* ASC = altered states of consciousness; IPIP = International Personality Item Pool.

* Measures collected but not analyzed in the current article, and will be reported elsewhere (along with EEG and eye-tracking data).

**Table 2.** Experiment 1: Visual phenomenology.

Category	Description	Example responses	*N* (proportion)	%
Elementary imagery	Observation of various shapes and patterns.	Spots, dots, paint splatters, lines, stripes, streaks	26 (.96) N:8 (1) B:9 (.9) W:9 (1)	96.3
Dynamic percepts	Descriptions of moving patterns, elements or shapes.	Twisting lines moving around, two figures turning around each other/dancing, abstract things that moved (network, veins of eyes)	12 (.44) N:4 (.5) B:2 (.2) W:6 (.67)	44.4
Complex imagery	Observation of more complex, defined imagery	Hands/oven mitts that are taking something out of the oven, shadows of people, person (like a ghost/shadow), face main character of Breaking Bad (Walter White), lion's head, fish swimming by, sails of ships	15 (.56) N:3 (.37) B:5 (.5) W:7 (.78)	55.6
Nature	Descriptions of nature elements	Rain, cobwebs, heavy rain, storm, water, bird, mouse	16 (.59) N:6 (.75) B:6 (.6) W:4 (.44)	59.3
Changing intensity	Descriptions of lighter or darker red	Light orange/yellow concentrated in one spot but disappeared when looking at it, changes in light, lighter and darker parts; not equal, changed the whole time, left side, dark at the bottom; shadows/dark toward ending, different intensities of red, higher contrast with other dark spots	13 (.48) N:2 (.25) B:7 (.7) W:4 (.44)	48.1

*Note.* The number of occurrences per noise condition are denoted with N for no-noise, B for brown-noise, and W for white-noise. Figure licensed under CC BY 4.0 by the authors.

*Source*. Retrieved from https://doi.org/10.6084/m9.figshare.28070858.v1.

**Table 3.** Experiment 2: Visual phenomenology.

Category	Description	Example responses	*N* (proportion)	%
Elementary imagery	Observation of various shapes and patterns.	Spots, dots, stripes, lines, light streaks, vague shadows, clouds	37 (.90) N:11 (.85) B:13 (.93) W:13 (.93)	90.2
Dynamic percepts	Descriptions of moving patterns, elements or shapes.	Black dots on screen almost moving up, tadpole swimming up and down, pulsating, cloud-like things moving, shark swimming by, blue flash falling down as a tear	39 (.95) N:13 (1) B:13 (.93) W:13 (.93)	95.1
Complex imagery	Observation of more complex, defined imagery	Person, boat, dolphins, apple, sharks, turtles	24 (.58) N:7 (.54) B:10 (.71) W: 7 (.5)	58.5
Nature	Descriptions of nature elements	Trees, water, storm, rain, animals, ripples or water in lake, flying birds, cows	25 (.61) N:9 (.69) B:9 (.64) W:7 (.5)	61

*Note.* The number of occurrences per noise condition are denoted with N for no-noise, B for brown-noise, and W for white-noise. Figure licensed under CC BY 4.0 by the authors.

*Source*. Retrieved from https://doi.org/10.6084/m9.figshare.28070882.v1.

The only consistent category that came out of the inductive content analysis, in all three experiments, was water-related hallucinations. Other reported sounds were quite random and less frequent, such as tooting, music, ticking, zooming etc. Tables 5, 6, and 7 contain the water-related auditory hallucinations reported in Experiments 1, 2, and 3, respectively.

**Table 4.** Experiment 3: Visual phenomenology.

Category	Description	Example responses	*N* (proportion)	%
Elementary imagery	Observation of various shapes and patterns.	Dots, stripes, geometrical shapes, undefined shapes	60 (.91) N:7 (.7) B:25 (.93) W:28 (.96)	90.9
Dynamic percepts	Descriptions of moving patterns, elements or shapes.	Shimmering like aquarium, explosion of dots, pulsating things, dynamic paint spreading in water, glistening, shapes getting smaller, colors fading away, vibration, noise, moving clouds, moving spots, moving smoke	40 (.61) N:6 (.6) B:16 (.59) W:18 (.62)	60.6
Complex imagery	Observation of more complex, defined imagery	Flowers, faces, panda bear, dinosaur, Mickey Mouse	34 (.51) N:6 (.6) B:12 (.44) W:16 (.55)	51.5
Nature	Descriptions of nature elements	Forest, seeing blossoming roses, grass-water-trees-like/nature, snowy landscape	34 (.47) N:4 (.4) B:12 (.44) W:18 (.62)	47
Vitreous opacities	Descriptions of seeing patterns that resemble broken ice, spider webs, retinal blood vessels, etc.	Like a crack in glass, dust on my eyes moving up and down, red veins, criss-cross like veins of an eye, volutes	14 (.21) N:5 (.5) B:4 (.15) W:5 (.17)	21.2

*Note.* The number of occurrences per noise condition are denoted with N for no-noise, B for brown-noise, and W for white-noise. Figure licensed under CC BY 4.0 by the authors.

*Source*. Retrieved from https://doi.org/10.6084/m9.figshare.28070897.v1.

**Table 5.** Experiment 1: Auditory hallucinations.

Category	Description	Example responses	*N* (proportion)	%
Water-related hallucinations	Hearing water-related sounds.	Associating noise with rain/storm, water because it sounds like the noise, seeing and hearing rain at the same time, heavy rain/storm, noise changed into recognizable sounds of water so I imagined a very vivid image of waterfall with nature around, waterfall whilst hearing water	6 (.22) N:0 (0) B:2 (.2) W:4 (.44)	22.2

*Note.* The number of occurrences per noise condition are denoted with N for no-noise, B for brown-noise, and W for white-noise. Figure licensed under CC BY 4.0 by the authors.

*Source.* Retrieved from https://doi.org/10.6084/m9.figshare.28070807.v2.

**Table 6.** Experiment 2: Auditory hallucinations.

Category	Description	Example responses	*N* (proportion)	%
Water-related hallucinations	Hearing water-related sounds.	Hearing rain, heard waterfall, nature sounds like being in rain forest, at blue I thought I heard water falling whilst seeing swimming pool, noise reminded me of water so I heard water falling (drops)	4 (.98) N:0 (0) B:1 (.07) W:3 (.21)	9.8

*Note.* The number of occurrences per noise condition are denoted with *N* for no-noise, *B* for brown-noise, and *W* for white-noise. Figure licensed under CC BY 4.0 by the authors.

*Source.* Retrieved from https://doi.org/10.6084/m9.figshare.28070864.v1.

The reports pertaining to multisensory integration are shown in Tables 8, 9, and 10 for Experiments 1, 2, and 3, respectively. As expected, based on the reported auditory hallucinations, the only category that came forth out of the inductive content analysis was once again water-related hallucinations in all three experiments.

**Table 7.** Experiment 3: Auditory hallucinations.

Category	Description	Example responses	*N* (proportion)	%
Water-related hallucinations	Hearing water-related sounds.	Sound of the sea, storm/lightning, rain, waves, zooming of sea, thunder, drips of water, waterfall	9 (.14) N:1 (.1) B:7 (.26) W:1 (.03)	13.6

*Note.* The number of occurrences per noise condition are denoted with N for no-noise, B for brown-noise, and W for white-noise. Figure licensed under CC BY 4.0 by the authors.

*Source.* Retrieved from https://doi.org/10.6084/m9.figshare.28070885.v1.

**Table 8.** Experiment 1: Multisensory integrated hallucinations.

Category	Description	Example responses	*N* (proportion)	%
Water-related hallucinations	Hallucinations related to water in multiple modalities	Seeing a waterfall while hearing water and seeing a rain forest while smelling rain, seeing and hearing rain at the same time	2 (.74) N:0 (0) B:0 (0) W:2 (.25)	7.4

*Note.* The number of occurrences per noise condition are denoted with N for no-noise, B for brown-noise, and W for white-noise. Figure licensed under CC BY 4.0 by the authors.

*Source.* Retrieved from https://doi.org/10.6084/m9.figshare.28070837.v1.

**Table 9.** Experiment 2: Multisensory integrated hallucinations.

Category	Description	Example responses	*N* (proportion)	%
Water-related hallucinations	Hallucinations related to water in multiple modalities	Water, wavy, at dark blue while hearing rain, at blue I thought I heard water falling whilst seeing swimming pool	2 (.05) N:0 (0) B:1 (.07) W:1 (.07)	4.9

*Note.* The number of occurrences per noise condition are denoted with N for no-noise, B for brown-noise and W for white-noise. Figure licensed under CC BY 4.0 by the authors.

*Source.* Retrieved from https://doi.org/10.6084/m9.figshare.28070873.v1.

Our inductive content analysis rendered an additional association pertaining to the color of the visual field as participants recurrently specified the co-occurrence of water-like hallucinations when the color of the GF was blue as reported in a previous paper in which we focused on clarifying the phenomenology and terminology of the GF effect from a visual perception viewpoint (Pistolas & Wagemans, 2025). As such, water-related imagery was reported during the blue GF by 11 out of 41 (27%) participants in Experiment 2 and 11 out of 66 (17%) participants in Experiment 3. Examples of descriptions entail “at blue I saw water,” “fish and ocean at blue,” “at blue a light source like in an ocean, there is light above the water that became bigger and bigger,” “blue was like being in the sea,” “sharks and turtles, when I saw this, the noise sounded like sea,” and “a shark swimming by at blue.”

Chi-squared tests of independence showed that there was no significant association between noise condition and visual hallucinations. We found *X*^2^ (2, *N* = 27) = 0.22, *p* = .90 for Experiment 1, *X*^2^ (2, *N* = 41) = 2.21, *p* = .33 for Experiment 2, and *X*^2^ (1, *N* = 56) = 0.95, *p* = .33 for Experiment 3.^2^ Table 11 contains the number of participants reporting visual hallucinations per condition.

**Table 10.** Experiment 3: Multisensory integrated hallucinations.

Category	Description	Example responses	*N* (proportion)	%
Water-related hallucinations	Hearing water-related sounds.	At blue the sound was more like the sea, when seeing normal colors I was more aware of noise, at blue seeing swimming fish or bird together with hearing sea/water/wind, heard sound of sea: at blue, with fish, knew it was noise but made sea of it, at blue and waterfall my senses came together and it felt like floating	10 (.15) N:1 (.1) B:6 (.22) W:3 (.1)	13.6

*Note.* The number of occurrences per noise condition are denoted with N for no-noise, B for brown-noise and W for white-noise. Figure licensed under CC BY 4.0 by the authors.

*Source.* Retrieved from https://doi.org/10.6084/m9.figshare.28070894.v1.

Concerning the auditory hallucinations, Chi-squared tests of independence yielded no significant associations between noise condition and auditory hallucinations in Experiments 1 and 2, with respectively, *X*^2^ (2, *N* = 27) = 4.49, *p* = .11 and *X*^2^ (2, *N* = 41) = 4.62, *p* = .10. A Chi-squared test of independence on the data of Experiment 3, however, did show a significant difference between the two noise conditions in auditory hallucinations (*X*^2^ (1, *N* = 56) = 5.18, *p* = .02). Note that there is a trend in the same direction for Experiments 1 and 2, unlike the results of the visual hallucinations in the previous section, for which all Chi-square values are smaller than 1. A post-hoc analysis in which we omit the no-noise condition in Experiments 1 and 2 yields similar results (*X*^2^ (1, *N* = 27) = 0.43, *p* = .52 and *X*^2^ (1, *N* = 41) = 0, *p* = 1), indicating that the omission of the no-noise condition is unlikely to explain the significant difference between the noise conditions in Experiment 3. Table 12 contains the number of participants reporting auditory hallucinations per condition.

**Table 11.** Number of participants reporting visual hallucinations per condition in each experiment.

Experiment	No noise	Brown noise	White noise
1	8 (1)	10 (1)	9 (1)
2	12 (.92)	14 (1)	14 (1)
3	8 (.8)	27 (1)	28 (.96)

*Note.* Proportions are included between parentheses. Figure licensed under CC BY 4.0 by the authors.

*Source.* Retrieved from https://doi.org/10.6084/m9.figshare.28070900.v1.

**Table 12.** Number of participants reporting auditory hallucinations per condition in each experiment.

Experiment	No noise	Brown noise	White noise
1	0 (0)	3 (.3)	4 (.44)
2	0 (0)	4 (.29)	4 (.29)
3	2 (.2)	11 (.41)	4 (.14)

*Note.* Proportions are included between parentheses. Figure licensed under CC BY 4.0 by the authors.

*Source.* Retrieved from https://doi.org/10.6084/m9.figshare.28070909.v1.

Due to the discrepancy in number of reported auditory hallucinations compared to visual hallucinations, the feasibility of a robust quantitative analysis of multisensory hallucinations was limited. Therefore, we asked a few additional questions during the experiment as an attempt at getting a better sense of why our participants were not experiencing as many auditory hallucinations (see the method section for the specifics). During the inductive content analysis of the additional questions of the interview data, we identified the recurring report of a unimodal focus on the visual aspect with 35 out of 66 (53%) participants reporting that they were primarily focused on the visual. A few example responses entailed “I was fixated on what I thought I saw,” “More focused on visual,” “I expected to only experience visual hallucinations, no auditory hallucinations,” “Attention on my eyes and focus on visual,” and “Very much focused on what I saw but was always aware of noise.” Another interesting recurring report entailed switching of attention between the visual and auditory sensory modalities with 22 (35%) participants reporting this. As such, some example responses were “Hearing heartbeat and breathing but did not pull me away from the visual,” “Switching of focus between modalities: visual, auditory and olfactory,” “Switching between modalities when one is more intense than other,” “Switching between visual and auditory,” and “Attention switched between modalities, not able to focus on 3 things simultaneously (visual, auditory, olfactory).” A related recurring report that we decided to code separately entailed the waxing and waning of perceived noise. Sixteen (24%) participants reported this experience along the lines of “I did not hear anything, after a few minutes the noise disappeared more into the background,” “I found the noise relaxing, focus on noise disappeared, it felt like I was not hearing any noise, but sometimes awareness came back that there was sound,” “Sound of noise disappeared to background, I was only aware of sound when there were background sounds,” and “Noise fell away to background.”

## Discussion

In this study, we aimed to comprehensively explore the phenomenology of multisensory hallucinations induced by multimodal GF stimulation, with a particular focus on the nature of reported percepts and multisensory integration. Our findings reveal that hallucinations were primarily perceived in the visual modality, with auditory hallucinations being less frequent overall. Interestingly, among the reported auditory experiences, water-related sounds were consistently highlighted, particularly in brown noise conditions. Furthermore, our qualitative analysis provided insights into the dynamics of unimodal focus and instances of attention-switching within the multimodal GF experience.

While building upon prior research into GF-induced experiences, including the work by Schmidt and Prein (2019), our study specifically aimed to assess the phenomenology across all sensory modalities, by explicitly instructing participants to report anything they might see, hear, feel, or smell, in contrast to a primary focus on the auditory component in some previous research. A notable discrepancy between our data and other multimodal studies (Lloyd et al., 2012; Schmidt & Prein, 2019) is the few auditory hallucinations our participants experienced. A potential explanation for this discrepancy could be the central role of instructions given to participants preceding the experiment. Schmidt and Prein (2019) instructed participants to “report any notice of auditory structure other than noise during the Ganzfeld exposure and indicated such events via button-press.” Similarly, Lloyd et al. (2012) instructed participants to focus on the white noise. This type of instruction might have elicited a larger focus on the auditory component than in our study, in which we instructed participants to report anything they might see, hear, smell, and feel during the experience for which there was no physical basis given that they were exposed to a homogeneous visual field and homogeneous noise or no noise. In turn, given that we framed our study as a light installation experience, this might have implicitly directed participants’ focus more on the visual component. Both Schmidt and Prein and Lloyd and colleagues also raised the role of instruction as a potential explanation for why they did not find more visual hallucinations, which is expected under GF literature based on a relatively large body of literature on the visual phenomenology of the GF effect (Pütz et al., 2006; Wackermann et al., 2002). Alternatively, the fact that the English language has a broader and more diverse lexicon of visual words compared to words related to other modalities (Winter et al., 2018) could potentially explain why participants do not report as many auditory hallucinations, especially vague ones for which we do not have a “shape equivalent” for example.

Beyond these considerations regarding instructions and linguistic factors, the properties of the auditory stimulus itself also play an important role in the emergence and nature of GF-induced hallucinations. The role of noise in the emergence of hallucinations, in particular, has been explored by Billock and Tsou (2007) who reported certain TV noise patterns induced hallucinatory percepts but not others. Their subsequent investigations, employing 1/f^n^ spatiotemporal noise (similar to brown noise), revealed that the level of noise contrast required to trigger these hallucinations was dependent on the spatial and temporal exponents. Notably, 1/f noise proved nearly optimal for this effect. They posited that this phenomenon might be linked to a variant of stochastic resonance, facilitating pattern formation.

The water-related auditory hallucinations, which formed the only frequently recurring category rendered by the inductive content analysis, may point to a potential suggestibility of the noise per participants’ own descriptions. As such, participants described these water-like auditory hallucinations to stem from the type of noise they were hearing. One participant specified this further with “noise reminded me of water so I heard water falling, drops.” This awareness of the suggestibility of the noise is particularly interesting given the fact that only one participant across all three experiments reported water-like auditory hallucinations in the no-noise condition. This person mentioned “hearing storm, lightning, like it was raining,” and further elaborated that “blue was cold, like being in a storm the whole time,” instigating the belief that the color blue might have also played a suggestive role in the experience of water-like auditory hallucinations given that they mentioned a connection between the storm and hearing rain, and between blue and hearing a storm. Indeed, our qualitative data indicates a similar suggestibility between the color of the GF and the experienced imagery given the recurring category rendered by the inductive content analysis in which responses that mentioned the association between the blue GF and the experience of water-like hallucinations were coded.

The no-noise condition was not found to differ in occurrence of visual hallucinations, contrasting previous reports that spontaneous, dream-like hallucinations seemed more frequent in the multimodal GF compared to the unimodal GF (Miskovic et al., 2019). Our results do indicate that brown noise evokes more auditory hallucinations. Considering its lower frequency and therefore increased resemblance to water and waves sounds, this could potentially reflect the suggestibility of the brown noise sounding similar to water, or ocean waves. In line with this finding, Schmidt and Prein (2019) found a nonsignificant trend for more auditory hallucinations in the brown-noise condition compared to white- and violet-noise conditions. In addition, Rogowitz and Voss (1990) reported that noise patterns with fractal statistics that resembled the statistics of natural images enhanced the emergence of imagery. Previous studies that employ a multimodal GF have not elaborated on how multimodal GF stimulation differs from unimodal GF stimulation and our data does not suggest there to be a clear difference in the emerging visual or auditory hallucinations between the no-noise and noise conditions.

The unimodal focus on the visual component that we identified from the inductive content analysis, is in line with the general belief that the processing of visual information is preferred over the processing of information from other sensory modalities, termed visual dominance (Cohen et al., 2009; Klein & Posner, 1974). Besides this general tendency, framing the study as a light installation experience might also have implicitly drawn the focus to the visual component slightly more despite our clear instructions to focus on all senses, that is, visual, auditory, olfactory, and bodily sensations.

Concerning the switching of attention between the visual and auditory modalities, there does not seem to be a consensus in the current state of the literature pertaining to multisensory integration as it is not clear whether simultaneous attention to both visual and auditory modalities is necessary for audio-visual integration (Evans, 2020). Our results offer mixed indications with some participants specifying that they could not focus on all the different modalities at the same time, while others did mention that all the information in the different senses came together as a whole in which they, for instance, experienced a storm with the corresponding visual and auditory characteristics of thunder, rain, wind, etc. While the latter describes the emergence of multisensory integration, the former might be indicative of a procedural artifact in which participants might be disconnected from the experience due to the instruction to use the dial to report these emerging phenomena. In turn, this might inhibit them from being fully immersed in the MMGF with further inhibition of the emergence of spontaneous multisensory hallucinations as a result.

However, the “disappearance of noise” as participants sometimes mentioned, denoting the perception of a disappearance of the homogeneous noise, further supports the notion of visual dominance. Another potential explanation could be that this disappearing of the noise might be the auditory equivalent of the frequently reported black-outs under GF stimulation, characterized by a darkening of the visual field to a considerable extent with participants often specifying that their visual field turns completely black. The emergence of visual black-outs is thought to be related to the attenuation of steady signals and unattenuated passing of transient signals by the visual system as proposed by Sparrock (1969).

The emergence of multisensory integrated hallucinations has not frequently been reported in past research. Lloyd et al. (2012) referred to multisensory experiences as the cases when visual percepts were reported to co-occur with semantically related auditory perceptions but did not elaborate on the frequency of these reports. These comprised most of the visual percepts that were reported in their study. Note that in their study, strikingly fewer visual hallucinations were reported compared to other GF studies in which participants were not instructed to focus on the auditory component. Overall, our data suggests that multisensory integrated experiences do not occur frequently under GF stimulation. Notably, no participants in the no-noise condition of Experiments 1 and 2 and only one participant in the no-noise condition in Experiment 3 reported a multisensory integrated experience.

In conclusion, our data seem to support the notion of visual dominance in human perception and more specifically, the GF experience seems to draw participants’ attention more to the visual aspects than the auditory ones. We doubt that referring to the experience as a light installation experience fully explains this direction of the focus on the visual component, given that we emphasized that participants might experience hallucinatory experiences in all sensory modalities and specifically named visual sensations as well as auditory, olfactory and bodily sensations. Rather, we suspect that the fact that we rely more on visual information in everyday life to anticipate our surroundings (Pike et al., 2012) transfers to this observed unimodal focus on the visual component in our GF set-ups. Future research on this topic might benefit from comparing both unimodal visual and auditory GF stimulation with either the instruction to focus on the visual aspect, the auditory aspect or both.

## Notes

1. Although we aimed at asking participants the same questions, we allowed for some degrees of freedom during the interview to allow for follow-up questions whenever it was not clear what participants meant and to better assess aspects that were mentioned repeatedly by several participants.

2. Note that df = 1 and N = 56 because we omitted the no-noise condition in Experiment 3.
